# Association of nitrite inhalants use and unprotected anal intercourse and HIV/syphilis infection among MSM in China: a systematic review and meta-analysis

**DOI:** 10.1186/s12889-020-09405-x

**Published:** 2020-09-10

**Authors:** Yonghui Zhang, Rantong Bao, Sequoia I. Leuba, Jiaming Li, Hongyi Wang, Jing Zhang, Zhenxing Chu, Wenqing Geng, Yongjun Jiang, Junjie Xu

**Affiliations:** 1 NHC Key Laboratory of AIDS Immunology (China Medical University), Department of Laboratory Medicine, The First Affiliated Hospital of China Medical University, No 155, Nanjing North Street, Heping District, Shenyang, 110001 Liaoning Province China; 2 National Clinical Research Center for Laboratory Medicine, The First Affiliated Hospital of China Medical University, Shenyang, 110001 China; 3 Key Laboratory of AIDS Immunology of Liaoning Province, The First Affiliated Hospital of China Medical University, Shenyang, 110001 China; 4 Key Laboratory of AIDS Immunology, Chinese Academy of Medical Sciences, Shenyang, 110001 China; 5 Collaborative Innovation Center for Diagnosis and Treatment of Infectious Diseases, 79 Qingchun Street, Hangzhou, 310003 China; 6 Department of Epidemiology, University of North Carolina at Chapel Hill, Chapel Hill, NC USA

**Keywords:** Nitrite inhalants, Men who have sex with men (MSM), HIV, Syphilis

## Abstract

**Background:**

Nitrite inhalant use is very common among men who have sex with men (MSM) in China. However, there is lack of national representative data on use among Chinese MSM, and the mechanism of how nitrite inhalant use impacts HIV infection in MSM is unclear. This meta-analysis aims to understand the characteristics of Chinese MSM nitrite inhalant users and to determine the associations between nitrite inhalant use and sexual behaviors, the prevalence of HIV, and the prevalence of syphilis.

**Methods:**

We searched PubMed, Web of Science, Chinese National Knowledge Infrastructure, Chinese Wanfang Data, and VIP Chinese Journal Database for relevant literature published from January 1985 to December 2017.

**Results:**

Fifteen eligible studies, with a total of 18,981 Chinese MSM participants, were included. Compared with nitrite inhalant nonusers, users were more likely to be younger, have a higher level of education, and seek sexual partners using the Internet. Nitrite inhalant users were more likely to engage in high-risk sexual behaviors, including condomless anal intercourse (odds ratio [OR] = 1.33), group sex (OR = 2.26), and commercial intercourse (OR = 1.50). Nitrite inhalants users had a higher prevalence of HIV (OR = 1.83), higher prevalence of syphilis (OR = 1.38) and had higher lifetime HIV testing rates (OR = 1.33) compared with nonusers (each *p* < 0.05).

**Conclusions:**

Nitrite inhalant users have higher HIV and syphilis prevalence by increasing levels of high-risk sexual behaviors. China should expand HIV testing, treatment as prevention (TasP), and Pre-exposure prophylaxis (PrEP) among MSM, especially among nitrite inhalants using MSM, to reduce their risk of HIV infection and transmission.

## Background

Men who have sex with men (MSM) are disproportionately affected by HIV; in 2017, 18% of incident HIV infections globally were among MSM [1]. In China, the proportion of newly diagnosed HIV infections contracted through male same-sex intercourse rapidly increased from 17.4% in 2011 [2] to 27.6% in 2016 [3]. Young MSM may be disproportionately affected as 47.0% of MSM diagnosed with HIV from 2006 to 2015 in China were between 20 and 29 years old [4], the number of HIV cases among Chinese university students increased by 35% annually from 2011 to 2015 [5], and in 2015, 82.6% of those diagnosed were infected through male-to-male sexual intercourse [6]. Overall, the HIV prevalence among MSM students increased from 3.0% during 2003–2006 to 5.2% during 2012–2016 [7, 8]. Therefore, determining risk factors for HIV acquisition among Chinese MSM, specifically young MSM, is urgently needed to develop effective HIV prevention and control strategies.

Nitrite inhalers are widely used among MSM to help enhance sexual pleasure by dilating capillaries and relaxing anal sphincters thus, reducing pain associated with anal sex. The use of nitrite inhalants as a recreational drug was initially reported in gay communities of developed countries [9–11]; however, as nations undergo rapid economic development, the use of these drugs is expanding globally. Nitrite inhalants (amyl or butyl nitrites) are commonly known as “rush” or “poppers” in China and are becoming increasingly popular among the MSM community in China and other Asian countries [12–14]. In recent years, Chinese MSM have increasingly purchased nitrite inhalants online and the proportion of lifetime nitrite inhalant use among Chinese MSM ranges from 10.6 to 53.6% [15, 16].

Despite the growing availability of nitrite inhalants and the well-documented association between nitrite inhalant use and HIV risk, the association between nitrite inhalants and HIV infection in Chinese MSM is unclear. Previous research found conflicting results on the relationship between nitrite inhalants use among Chinese MSM and HIV infection or HIV-related high-risk behaviors [17–20]. These previous studies were mostly short-term, small, and single-site thus limiting the extrapolation of their findings. Besides, the mechanism of how nitrite inhalants use impacts the risk of HIV infection is not well understood. Evidence from other settings suggest nitrite inhalants have a consistent and well-documented association with HIV seroconversion [21]. To address this gap in knowledge, we systematically reviewed articles to compare the demographics, sexual behaviors, prevalence of HIV, prevalence of syphilis, and HIV testing behaviors of Chinese MSM who use nitrite inhalants. These study findings could help support the development of targeted interventions promoting HIV prevention strategies among Chinese MSM nitrite inhalant users.

## Methods

### Literature search

This systematic review and meta-analysis followed the PRISMA guidelines [22] (Supplementary Table 1), and completed the systematic review registration (PROSPERO registration number: CRD42018104538). The databases identified for this review were PubMed, Web of Science, Chinese National Knowledge Infrastructure, Chinese Wanfang Data, and VIP Chinese Journal Database. The search included all studies published from January 1985 to December 2017. The reference lists of admissible articles were also reviewed for additional relevant studies. Keywords used in the database searches were as follows: (“men who have sex with men” OR “MSM” OR “homosexual” OR “gay”) AND (“HIV”) AND (“poppers” OR “Amyl “OR “butyl nitrites” OR “nitrite inhalants”) AND (“China” OR “Hong Kong” OR “Taiwan” OR “Macau”).

### Inclusion and exclusion criteria

Studies were included in the meta-analysis if they reported the proportion of nitrite inhalant use among MSM in China and if they were published in English or Chinese. Studies were excluded for the following reasons: (1) they were duplicate articles; (2) the study subjects were not MSM; (3) the study included only HIV-positive MSM; (4) the study was in an unrelated area or a review article; (5) the study did not specifically report the proportion of nitrite inhalants use; or (6) the study targeted drug abusers. For the same study published across multiple articles, the most comprehensive article was included in the meta-analysis.

### Data extraction

Subjects who reported using nitrite inhalants within the past year of study enrollment were classified as nitrite inhalants users, and subjects were recruited through Internet, community mobilization, peer recruitment, and other channels. All but one of the studies were cross-sectional; one study was prospective.

Two reviewers (YZ and RB) independently extracted the following information from the eligible studies: (1) basic information (i.e., first author, publication year, study location, study periods, recruitment methods, sample size, and recall window); (2) the proportion of nitrite inhalants users and the proportion of nitrite inhalants nonusers; (3) the prevalence of HIV infection; (4) the prevalence of current syphilis infection; (5) behavioral information (e.g., the main way to seek sexual partners, condomless anal intercourse (CAI) with males, anal sexual role, number of male sex partners, group sex, and commercial sexual behavior); and (6) HIV testing history. Any disagreement between the two reviewers was resolved by another investigator (JX).

### Quality assessment

The quality of the studies was assessed using the quality assessment tool for systematic reviews of observational studies (QATSO) score [23], a quality assessment checklist that has been validated for examining HIV prevalence and HIV high-risk behaviors among MSM (Supplementary Table 2). Items were scored as 1 (“yes”), 0 (“no”), and NA (“not applicable”). To be included in this meta-analysis studies were required to have total quality scores of ≥33% (where: poor = 0% ~ 32%; satisfactory = 33–66%; and good = 67–100%). Quality assessment scores are provided in Supplementary Table 3.

### Statistical analysis

Demographics and sexual behaviors were compared between nitrite inhalants users and nonusers using the chi-squared test. Where applicable, meta-analyses were performed to calculate the pooled proportion of nitrite inhalants use, the pooled prevalence of HIV infection, the pooled prevalence of current syphilis infection, the pooled proportion of HIV-related high-risk sexual behaviors, and the pooled proportion of lifetime HIV testing. Pooled odds ratios (pooled ORs) and their 95% confidence intervals (CIs) were reported for these meta-analyses.

Statistical heterogeneity was quantified using the *I*^*2*^ index, with values of 25, 50, and 75% indicating low, medium, and high heterogeneity, respectively. If significant homogeneity was detected (*I*^*2*^ > 50%), random effect models were used to calculate the pooled odds ratios; otherwise, fixed-effect models were used. We assessed the influence of each study on overall estimates by conducting sensitivity analyses where each study was sequentially individually omitted, to determine any bias attributable to the omitted study. We investigated publication bias using Egger’s test. All statistical tests were performed using R software (version 3.4.3). Meta-analyses were conducted using R software with Meta package (version 4.9–2). A two-tailed *P-*value of less than 0.05 was considered statistically significant.

## Results

### Overview of studies

**Fig. 1:**
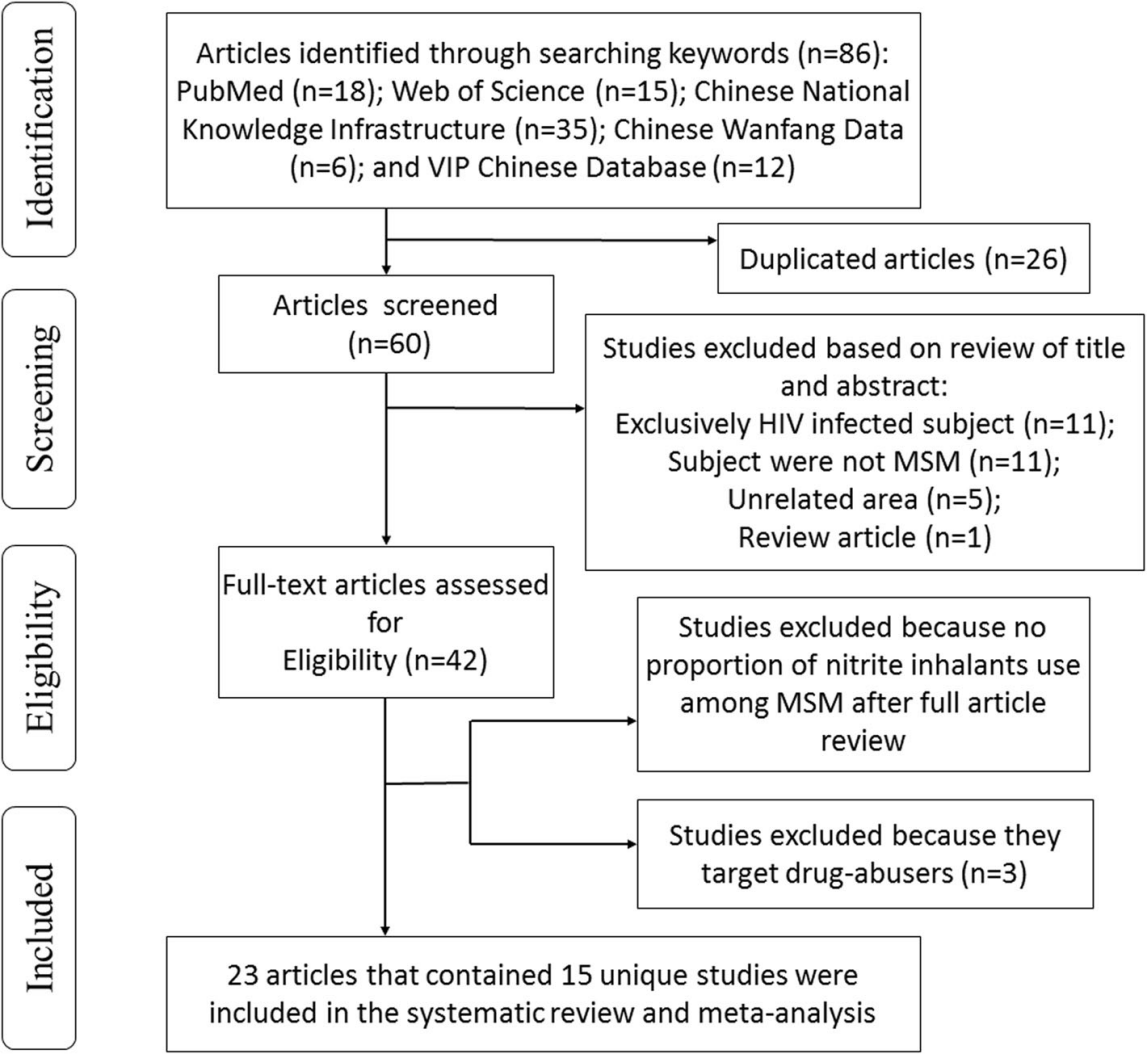
Flow chart of literature search for the systematic review and meta-analysis

As shown in Fig. 1, eighty-six relevant articles were identified from January 1985 to December 2017, of which 60 articles entered further screening, and 15 studies (seven published in English and eight in Chinese, spanning 18,981 MSM) were included in our systematic review and meta-analysis (Fig. 1). As shown in Supplementary Table 4, all studies restricted enrollment to participants aged 16 years or older. Most studies used the Internet to recruit participants (11/15, 73.3%).

### Demographics

As shown in Table 1, eight studies investigated the differences in age or educational level between MSM nitrite inhalants users and nonusers. Seven of these studies found a significant difference between the age of nitrite inhalants users and nonusers, of which six reported that nitrite inhalants users tended to be younger than nonusers (*P*-values for all < 0.05) [17, 19, 21, 24–27], while only one study reported that nonusers were younger than users (*P* = 0.028) [28]. In addition, all studies that investigated differences in education level found that nitrite inhalants users were more likely to have had college or higher education compared with nonusers, with six studies reporting statistically significant differences (*P*-values for all < 0.05) [17, 19, 24–27].

**Table 1:** Comparison of age and education level of MSM who use nitrite inhalants and of MSM who do not use nitrite inhalants in China

Study	Age			Education		
	Age category (years)	Nitrite inhalants users N(%)^a^	Nitrite inhalants nonusers N(%)^a^	Education level	Nitrite inhalants users N(%)^a^	Nitrite inhalants nonusers N(%)^a^
Chen et al. (2016) [24]	16–20	9 (7.4)	92 (13.1)^*^	Senior high school and below	4 (3.3)	29 (4.1)
	21–25	61 (50.0)	389 (55.3)	College/Bachelors	79 (64.8)	457 (65.0)
	26–30	52 (42.6)	222 (31.6)	Masters or PhD	39 (32.0)	217 (30.9)
Chu (2013) [17]	≤30	99 (82.5)	317 (62.8)^*^	Senior high school and below	64 (53.3)	326 (64.6)^*^
	> 30	21 (17.5)	188 (37.2)	College/Bachelors and above	56 (46.7)	179 (35.4)
Li (2014) [19]	≤28	417 (66.1)	1070 (49.2)^*^	Senior high school and below	276 (43.7)	1055 (48.5)^*^
	> 28	214 (33.9)	1105 (50.8)	College/Bachelors and above	355 (56.3)	1120 (51.5)
Li et al. (2014) [25]	< 25	49 (25.9)	33 (15.6)^*^	≤12 years education experience	49 (25.9)	93 (44.1)^*^
	≥25	140 (74.1)	178 (84.4)	> 12 years education experience	140 (74.1)	118 (55.9)
Wang et al. (2017) [26]	18–30	109 (71.7)	193 (53.9)^*^	Less than college	53 (34.9)	200 (55.9)^*^
	31–70	43 (28.3)	165 (46.1)	College and above	99 (65.1)	158 (44.1)
Xu et al. (2017) [27]	< 25	103 (41.9)	309 (38.9)^*^	Less than college	54 (22.0)	234 (29.5)^*^
	25–49	139 (56.5)	441 (55.5)	College and above	192 (78.0)	560 (70.5)
	≥50	4 (1.6)	44 (5.5)			
Zhang et al. (2016)^b^ [28]	18–34	831 (86.6)	2007 (76.5)^*^	Senior high school and below	191 (19.9)	818 (31.1)^*^
	35–75	129 (13.4)	618 (23.5)	College/Bachelors and above	770 (80.1)	1809 (68.9)
Zhao et al. (2017) [29]	< 20	36 (11.9)	170 (15.2)	Senior high school and below	66 (21.8)	303 (27.0)
	20–29	203 (67.0)	695 (62.0)	College/Bachelors	219 (72.3)	750 (66.9)
	≥30	64 (21.1)	256 (22.8)	Masters or PhD	18 (5.9)	68 (6.1)

^a^ Percentages may not add up to 100 because of rounding

^b^ This study has missing data for either age or educational level

Ten studies did not include any information on the age or on the education level of MSM who use nitrite inhalants and of MSM who do not use nitrite inhalants and thus were not included in this table

The comparison of the distribution of age or the distribution of education level between MSM who use nitrite inhalants and MSM who do not use nitrite inhalants were included in this table if the comparison was available in the study. Comparisons used the chi-squared test, and a *P*-value of less than 0.05 was defined as significant and marked with *

### Analysis of nitrite inhalants use

**Fig. 2:**
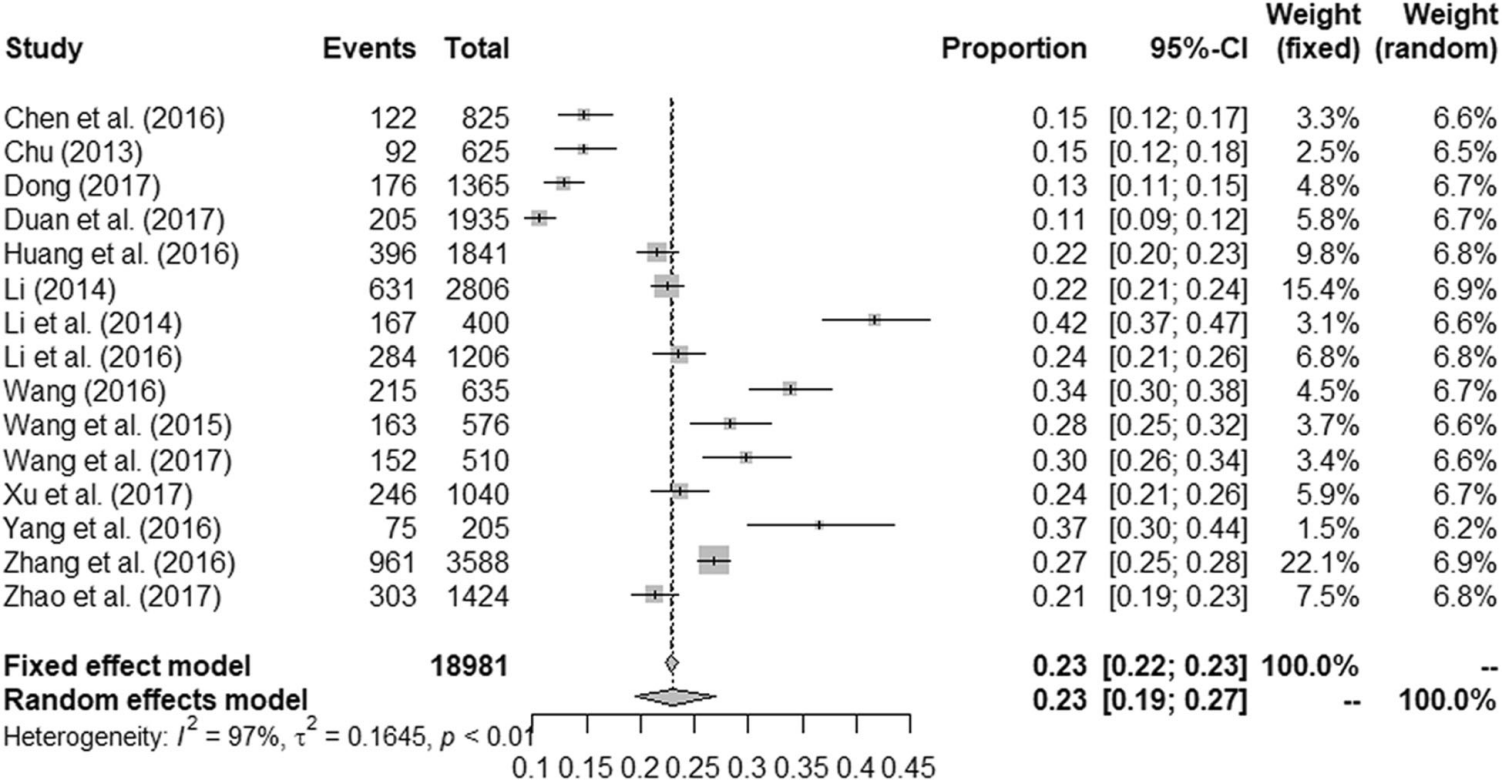
Forest plot of the proportion of MSM who use nitrite inhalants

A recent study from Ireland showed that 33% of MSM used nitrite inhalants in a nationwide cross-sectional study [29]. In the 15 studies included in this review, the pooled proportion of MSM who had ever used nitrite inhalants was 0.23 (95% CI: 0.19–0.27) (Fig. 2).

In order to examine possible differences in the proportion of nitrite inhalants users, subgroup analyses were conducted for the study period, study site, sample size, data collection method, recall window of nitrite inhalants use, and publication language (Table 2). Subgroup analysis by study period found that the proportion of nitrite inhalants users varied at different time intervals (27.7% from 2011 to 2013; 18.9% from 2014 to 2016). Studies conducted in northern China had a higher proportion of nitrite inhalants users compared to those conducted in southern China (26.0% vs. 22.4%). The proportion of nitrite inhalants users was higher when the sample size was small: 28.4% for studies with 1000 participants or fewer, and 20.3% for over 1000 participants. Furthermore, the proportion of nitrite inhalants users was slightly higher when data were collected using a self-administrated questionnaire than when data were collected using a face-to-face interview (25.2% vs. 23.1%). The proportion of nitrite inhalants users was higher when studies with a recall window of 12 months (31.4%); 3 months (23.7%); 6 months (22.0%). Finally, English-language publications reported a slightly higher proportion of nitrite inhalants users compared with those in Chinese (27.6% vs. 20.8%).

**Table 2:** Stratified meta-analyses of the proportion of MSM who use nitrite inhalants in China

Category	Subgroup	Number of studies	Proportion estimate (%) and 95% CI	Heterogeneity	
				*I*^*2*^	*P*-value
Study period	2011–2013	6	27.7 (21.8, 33.7)	95.8%	< 0.01
	2014–2016	7	18.9 (14.3, 23.5)	97.1%	< 0.01
Study site	Northern China	6	26.0 (21.1, 31.0)	95.9%	< 0.01
	Southern China	5	22.4 (14.6, 30.2)	97.7%	< 0.01
	Other^a^	4	22.5 (15.8, 29.2)	97.6%	< 0.01
Sample size	≤1000	7	28.4 (20.4, 36.3)	97.1%	< 0.01
	> 1000	8	20.3 (15.8, 24.8)	98.0%	< 0.01
Data collection method	Self-administrated questionnaire	6	25.2 (17.2, 33.3)	98.2%	< 0.01
	Face-to-face interview	9	23.1 (19.3, 26.9)	96.5%	< 0.01
Recall window of nitrite inhalants use (months)	3	6	23.7 (18.3, 29.2)	96.1%	< 0.01
	6	7	22.0 (16.4, 27.5)	98.1%	< 0.01
	12	2	31.4 (11.3, 51.5)	98.3%	< 0.01
Publication language	English	7	27.6 (20.0, 35.2)	98.5%	< 0.01
	Chinese	8	20.8 (16.9, 24.8)	96.0%	< 0.01

^a^ These studies were not conducted in one district; Zhao et al. (2017) was a nation-wide online survey, Wang et al. (2017) was conducted in Beijing and Nanjing, Dong (2017) was conducted in Shanghai and Tianjin, and Li (2014) was conducted in seven cities in China

### Sexual behaviors

Nine studies in this systematic review included information comparing sexual behaviors between nitrite inhalants users and nonusers (Table 3). Seven studies reported that the proportion of MSM who sought sexual partners through the Internet was significantly higher among nitrite inhalants users compared to nonusers (*P*-values < 0.05 for all) [15, 17, 19, 24–27]. Five studies found statistically significant differences for sexual role, as nitrite inhalants users tended to choose a receptive or versatile sexual role (*P*-values for all < 0.05) rather than insertive, relative to nonusers [15, 25–27, 30]; however, three other studies found no statistically significant differences in sexual role between nitrite inhalants users and nonusers [17, 19, 28]. Five studies found that nitrite inhalants users tended to have more male sexual partners compared with nonusers [15, 24, 26–28] (*P*-values for all < 0.05), and one study found that the number of casual sexual partners in the past 3 months was higher among nitrite inhalants users than nonusers (*P*-values for all < 0.05) [28].

**Table 3:** Venue for seeking sex partners/Sexual behaviors among MSM who use nitrite inhalants and among MSM who do not use nitrite inhalants in China

Study	Venue for seeking sex partners/Sexual Behaviors	nitrite inhalants users		nitrite inhalants nonusers		*P*-value
		N	%^a^	N	%^a^	
Wang et al. (2017) [26]	Sexual role					0.001
	Insertive	41	27.3	144	42.9	
	Receptive/Versatile	109	72.7	192	57.1	
	No. of sex partners, P6M					< 0.001
	< 2	28	18.4	136	38.0	
	≥2	124	81.6	222	62.0	
	CRAI, P6M	57	52.3	114	52.5	0.967
	Seeking male sex partners via the Internet	145	95.4	267	74.6	< 0.001
Zhao et al. (2017) [29]	Sexual role					0.013
	Insertive	93	30.7	431	38.4	
	Receptive/Versatile	210	69.3	690	61.6	
	Gay apps users^c^	220	72.6	604	53.9	< 0.001
Xu et al. (2017) [14]	Sexual role					0.003
	Insertive	65	26.4	291	36.6	
	Receptive/Versatile	181	73.6	503	63.4	
	No. of sex partners, P6M					< 0.001
	≤1	66	26.8	467	58.8	
	≥2	180	73.2	327	41.2	
	CAI with casual partners, P6M	83	33.7	152	19.1	< 0.001
	Venue for seeking sex partners					0.001
	Internet	202	82.1	570	71.8	
	Others^d^	44	17.9	224	28.2	
Duan et al. (2017) [15]	Had receptive sexual intercourse, P6M	128	62.4	821	47.5	< 0.001
	Multiple sex partners (≥2), P6M	157	76.6	828	47.9	< 0.001
	Seeking male sex partners by Internet	164	86.3	704	60.2	< 0.001
Zhang et al. (2016) [21]	Sexual role					< 0.001
	Insertive	268	30.0	863	40.9	
	Receptive/Versatile	626	70.0	1249	59.1	
	No. of male sex partners, P3M					< 0.001
	≤1	372	38.7	1507	57.4	
	≥2	589	61.3	1120	42.6	
	CRAI, P3M	298	31.0	547	20.8	< 0.001
	Most common venue for seeking male partners					< 0.001
	Internet	834	86.8	2004	76.3	
	Other^e^	127	13.2	623	23.7	
Chen et al. (2016) [24]	Sexual role, recently					0.800
	Insertive	44	36.1	262	37.3	
	Receptive/Versatile	78	63.9	441	62.7	
	No. of casual partners ≥1, P3M	68	55.7	310	44.1	0.017
	Multiple sex partners (≥2), P3M	45	36.9	188	26.7	0.023
Li et al. (2014) [25]	Had a casual sex partner, P3M	135	71.4	126	59.7	0.014
	Venue for seeking sex partners					< 0.001
	Internet	158	83.6	130	61.6	
	Others^f^	31	16.4	81	38.4	
Li (2014) [19]	Sexual role					0.238
	Insertive	186	29.5	695	32.0	
	Receptive/Versatile	445	70.5	1480	68.0	
	No. of partners, P6M					0.003
	≤3	372	59.0	1423	65.4	
	> 3	259	41.0	752	34.6	
	Seeking sex partners via the Internet	554	87.8	1470	67.6	< 0.001
Chu (2013)^g^ [17]	Sexual role					0.050
	Insertive	26	21.7	155	30.7	
	Receptive/Versatile	94	78.3	350	69.3	
	Seeking sex partners via the Internet	86	71.7	252	49.9	< 0.001

*N/A* Not available; “one for night”: one-night stands, *CRAI* Condomless receptive anal intercourse, *CAI* Condomless anal intercourse, P3M: in the past 3 months; P6M: in the past 6 months

^a^ The percent of non-missing observations. Percentages may not add up to 100 because of rounding

^b^ Venues for seeking sex partners included parks, public toilets, bars, clubs, and bathhouses

^c^ They had used gay smart phone-based sex-seeking applications in the last six months

^d^ Venues for seeking sex partners included parks, public toilets, bathhouses, bars, dance halls, and clubs

^e^ Venues for seeking male partners included parks, bathrooms, sauna and other public places

^f^ Venues for seeking sex partners included parks and bathrooms

^g^ This data was extracted from the baseline questionnaire of this study

**Fig. 3:**
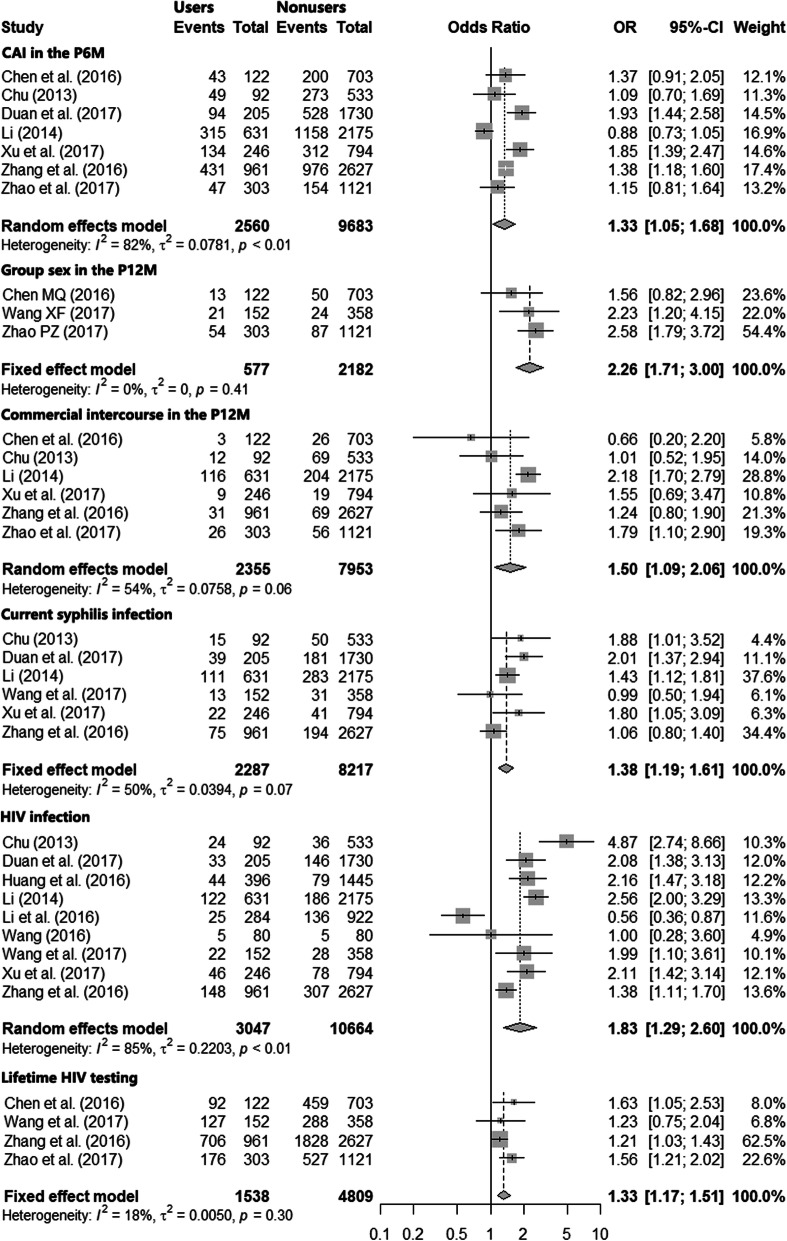
Forest plots comparing characteristics between MSM who use and those who do not use nitrite inhalants. Characteristics compared included, HIV prevalence, syphilis prevalence, and high-risk sexual behaviors. CAI, condomless anal intercourse; P6M, in the past 6 months

As shown in Fig. 3, nitrite inhalants users were more likely to have had CAI in the past 6 months (pooled percentages: 43.5% (1113/2560) vs. 37.2% (3601/9683); pooled OR = 1.33 (95% CI: 1.05–1.68); *I*^*2*^ = 82%; *P* < 0.010). Further, nitrite inhalant users were also more likely to have had group sex in the past 12 months (pooled percentages: 15.3% (88/577) vs. 7.4% (161/2182); pooled OR = 2.26 (95% CI: 1.71–3.00); *I*^*2*^ = 0%, *P* = 0.41), and to have taken part in commercial sexual behavior in the past 12 months (pooled percentages: 8.4% (197/2355) vs. 5.6% (443/7953); pooled OR = 1.50 (95% CI: 1.09–2.06); *I*^*2*^ = 54%; *P* = 0.06) compared with nonusers.

### Prevalence of HIV and prevalence of syphilis

Of the 15 studies included in this meta-analysis, three did not include information on HIV prevalence [16, 28, 31]; however, one of these three studies found that nitrite inhalants users had higher proportion of life-time sexually transmitted infection history compared to nonusers [28]. In addition to these three studies, another two studies collected data using online questionnaires and thus used self-reported testing results, rather than laboratory-based testing, for HIV or syphilis infection [30, 32]. We used only data from the 9 studies that used laboratory-based testing to compare HIV and syphilis prevalence rates among nitrite inhalants users and nonusers (Fig. 3). The prevalence of HIV (pooled percentages: 15.4% [469/3047] vs. 9.4% [1001/10664]; pooled OR = 1.83 [95% CI: 1.29–2.60]; *I*^*2*^ = 85%; *P* < 0.01) and the current syphilis prevalence (pooled percentages: 12.0% [275/2287] vs. 9.5% [780/8217]; pooled OR = 1.38 [95% CI: 1.19–1.61]; *I*^*2*^ = 50%; *P =* 0.07) were higher among nitrite inhalants users than nonusers.

### HIV testing behaviors

Four studies reported data on participants having ever been tested for HIV (Fig. 3) [25, 27, 28, 30]. Lifetime HIV testing was higher among nitrite inhalants users than nonusers (pooled percentages: 71.6% [1101/1538] vs. 64.5% [3102/4809]; pooled OR = 1.33 [95% CI: 1.17–1.51]; *I*^*2*^ = 18%; *P* = 0.30).

### Sensitivity analysis and publication bias

The pooled ORs remained similar in the sensitivity analysis, suggesting that the results were robust. Egger’s test suggested no evidence of publication bias in comparing the prevalence of HIV (*P* = 0.424) or that of syphilis (*P* = 0.907) between nitrite inhalants users and nonusers.

## Discussion

To the best of our knowledge, this is the first meta-analysis investigating the proportion of Chinese MSM who use nitrite inhalants. This study found that MSM who used nitrite inhalants were generally younger, had higher levels of education, had higher prevalence of HIV infection, and had higher prevalence of current syphilis infection, compared to nonusers. In terms of HIV-related high-risk sexual behaviors, nitrite inhalants users had higher number of sexual partners, were more likely to have CAI, group sex, commercial sex, and to have previously been tested for HIV, compared to nonusers. Our results summarize the characteristics, HIV-related high-risk behaviors, and HIV and syphilis prevalence among Chinese nitrite inhalants users. These study results increase our knowledge of the understanding of the effect of nitrite inhalants use on HIV related behavior and HIV epidemic among MSM in China.

In our meta-analysis, the pooled proportion of Chinese MSM who had ever used nitrite inhalants was 0.23 (95%CI: 0.19–0.27), which was lower than the proportions reported among MSM in New Zealand (0.37) [33], Canada (0.32) [34], and the United Kingdom (0.28) [35]. While the sale of nitrite inhalants has been banned in Canada and restricted in the United States and the European Union [36], it is not legally restricted in China, and nitrite inhalants can be purchased easily and cheaply through the Internet [37]. Since the Internet began to develop rapidly around 2010 in China, it also increased the scale of online e-commerce in the country, which promoted the usage rate of nitrite inhalants among MSM in China. Thus, the proportion of nitrite inhalants users among MSM in China may surpass that of Western countries in the future. Standardize the online sales process for nitrite inhalants, the Chinese government should develop, implement and enforce effective laws and interventions. While, we found the uneven geographic distribution of nitrite inhalants. Compared with southern China, North China had higher nitrite inhalants using rate among MSM population (33.1% vs. 22.8%). It indicate that the strategies to deal with inhalants use of MSM in different regions should be treated differently. The results of this meta-analysis suggest that the use of nitrite inhalants was associated with age and education level of users. Two studies found that a higher proportion of nitrite inhalants users were younger than 25 years old and had a college degree or above compared with nonusers [24, 26], suggesting future research must focus on nitrite inhalants use among MSM currently in college. A recent study conducted among MSM currently in college in Kunming, China found that 50% of MSM college students had ever used nitrite inhalants [38]. Younger and less sexually experienced MSM are more likely to feel pain during anal intercourse compared to older and more sexually experienced MSM, and thus are increasingly using nitrite inhalants to relax the anal sphincter to relieve this pain [39]; however, as using nitrite inhalants can also extend the duration of anal intercourse, nitrite inhalants use can thereby increase the probability of anal bleeding, consequently increasing the risk of HIV infection [40]. Although Chinese public health officials have developed intervention methods targeted towards young MSM, particularly college students, these measures primarily focus on disseminating HIV-related health knowledge, distributing condoms, and improving HIV testing. They do not include implementing interventions addressing the use of nitrite inhalants.

Our study also confirmed recent research findings that Chinese nitrite inhalants users were more likely to seek male sexual partners via the Internet than nonusers. This phenomenon may be due to the younger age of nitrite inhalants users compared with nonusers, and the fact that, in China, nitrite inhalants are easily and cheaply available via the Internet [14]. In addition, a previous systematic review found that computer-based interventions targeting illicit drug users can effectively reduce the frequency of recreational drug use [41]. Thus, future studies should focus on the development of Internet-based interventions, especially dating-applications-based interventions, to reduce their HIV infection and transmission risk. The Internet and dating-application platform should be well utilized to prompt HIV testing, treatment to prevention (TasP), and pre-exposure prophylactic (PrEP) to MSM, particularly to MSM using nitrite inhalants.

Our meta-analysis found that, nitrite inhalants users had a higher likelihood of currently being infected with syphilis and of HIV infection compared with nonusers in China. Studies conducted in France [42] and America [43] investigating the association between nitrite inhalants use and syphilis or HIV infection reported similar results. This meta-analysis strengthens the support for the association between nitrite inhalants use and syphilis or HIV infection.

Our results also reveal multiple mechanisms underlying the nitrite inhalants-induced increased risk of HIV acquisition. Based on the findings of our meta-analysis, we conclude that, compared with nitrite inhalants nonusers, users were more likely to have receptive anal intercourse, condomless anal intercourse, have more sexual partners, participate in group sex, and be involved in commercial sexual behavior. The per contact risk of HIV infection for condomless receptive anal sex is over three times that for condomless insertive anal sex (0.73% vs. 0.22%) [44]. In addition, the meta-analysis data indicate that nitrite inhalants users are more likely than nonusers to have CAI, thereby increasing their risk of HIV infection, similar to previously reported research [45]; however, in our meta-analysis, only three studies reported an association between condomless anal intercourse and an increased risk of HIV infection among MSM [15, 26, 30, 46]. This surprising outcome may be due to the small sample sizes in these studies, resulting in insufficient statistical power to discern differences between condomless anal intercourse behaviors between nitrite inhalants users and nonusers.

Our meta-analysis also found that nitrite inhalants users have more male sexual partners, particularly casual sexual partners, compared with nonusers. This result is consistent with a previous study conducted in Vancouver, Canada [34], and the finding that greater numbers of sexual partners are associated with an increased risk of HIV infection [47]. Similarly our results also indicated that the proportion of nitrite inhalants users who have group sex was 2.26 times that of nonusers, consistent with a study among MSM in London [48], and findings that participating in group sex increases the risk of HIV infection [49]. Finally, nitrite inhalants users may more likely to engage in commercial sexual behavior than nonusers in our meta-analysis. MSM who have ever sold sex to another male or bought sex from another male have higher levels of condomless anal intercourse and higher numbers of sexual partners in the past 6 months compared with those who have never been involved in commercial sex [46]. As these high-risk HIV-related factors associated with commercial sex are more common among MSM who engage in commercial sex, these MSM are therefore more likely to become infected with HIV, compared with MSM who do not participate in commercial sex. Overall, nitrite inhalants users have a higher prevalence of HIV relative to nonusers and, through increasing HIV-related high-risk behaviors. Hence, nitrite inhalants using behavior may lead to the high prevalence of HIV among MSM population.

Furthermore, our meta-analysis determined that the proportion of nitrite inhalants users who have had at least one HIV test in their lifetime was higher than that among nonusers. As nitrite inhalants, users are more likely to participate in HIV-related high-risk behaviors (i.e., multiple casual sexual partners, CAI, group sex, commercial sex, etc.) than nitrite inhalants nonusers. The prevalence of HIV higher among MSM who use nitrite inhalants, thus they are more engaged with preventative methods, including frequent HIV testing [50]. Since nitrite inhalants users have a higher prevalence of HIV infection, and our results found that nearly 70% of MSM nitrite inhalants users in China have ever been tested for HIV, compared with 49.7% of the general Chinese MSM population [51]. Future strategies should promote HIV testing among nitrite inhalants users who have never been tested for HIV before. Recent research has shown that crowd sourcing, conducted through the Internet, can promote first-time HIV testing among Chinese MSM [52]. Thus, future Internet-based strategies could be used to target nitrite inhalants users who have never been tested to promote HIV testing, thereby helping to mitigate the HIV epidemic among MSM.

A limitation of our meta-analysis is that the studies on nitrite inhalants use among MSM subjects were almost all cross-sectional investigations, except for only one prospective cohort study. Therefore, we were unable to access temporality and thus causality between the use of nitrite inhalants and increases in the risk of HIV acquisition among Chinese MSM. In addition, most of the studies were conducted in cities, thus the study results are not representative of Chinese MSM who live in rural areas. Finally, nitrite inhalants use was self-reported, and participants may have underreported use because of social desirability bias.

## Conclusions

This study provides a representative and comprehensive understanding of the prevalence of nitrite inhalants use among Chinese MSM. The use of nitrite inhalants increases the risk of HIV infection among MSM likely through increasing participation in high-risk sexual behaviors. To address the HIV epidemic, it is suggested to well utilize the expanded HIV testing, TasP, and PrEP among nitrite inhalants users to reduce their risk of HIV infection and transmission.

## Supplementary information

**Additional file 1: Table S1.** PRISMA Checklist. It contains a list of 27 items that describe what information to include.

**Additional file 2: Table S2.** Checklist of quality assessment. It contains 5 questions to evaluate prevalence studies, decision criteria and scores for each question.

**Additional file 3: Table S3.** Summary of quality assessment score of 15 studies. The 15 studies were assessed on the quality assessment which include each score of five questions and total score of each study.

**Additional file 4: Table S4.** Basic information about eligible studies. Basic information on 15 eligible studies such as study location, study periods, recruitment methods, survey method, eligibility of subjects, sample size, recall window (months), poppers use recall window (months).

## Data Availability

All data generated or analyzed during this study are included in this published article [and its supplementary information files].

## Abbreviations

MSM: Men who have sex with men
OR: Odds ratio
CI: Confidence intervals
CAI: Condomless anal intercourse
