# Supplementary material for: Association of nitrite inhalants use and unprotected anal intercourse and HIV/syphilis infection among MSM in China: a systematic review and meta-analysis
Source: BMC Public Health. 2020 Sep 10;20:1378. doi: 10.1186/s12889-020-09405-x (PMC7488293; doi:10.1186/s12889-020-09405-x)
Supplement: Supplementary file 1 — Additional file 1: Table S1. PRISMA Checklist. It contains a list of 27 items that describe what information to include. [file 12889_2020_9405_MOESM1_ESM.doc]

**Supplementary Table 1.** PRISMA Checklist

| **Section/topic** | **#** | **Checklist item** | **Reported Section #** |
| --- | --- | --- | --- |
| **TITLE** | | |  |
| Title | 1 | Identify the report as a systematic review, meta-analysis, or both. | Title |
| **ABSTRACT** | | |  |
| Structured summary | 2 | Provide a structured summary including, as applicable: background; objectives; data sources; study eligibility criteria, participants, and interventions; study appraisal and synthesis methods; results; limitations; conclusions and implications of key findings; systematic review registration number. | Abstract |
| **INTRODUCTION** | | |  |
| Rationale | 3 | Describe the rationale for the review in the context of what is already known. | Introduction – 1^st^ to 3^rd^ paragraphs |
| Objectives | 4 | Provide an explicit statement of questions being addressed with reference to participants, interventions, comparisons, outcomes, and study design (PICOS). | Introduction – 3^rd^ paragraph |
| **METHODS** | | |  |
| Protocol and registration | 5 | Indicate if a review protocol exists, if and where it can be accessed, and, if available, provide registration information including registration number. | PROSPERO: CRD42018104538 |
| Eligibility criteria | 6 | Specify study characteristics and report characteristics used as criteria for eligibility, giving rationale. | Method – Inclusion and Exclusion Criteria |
| Information sources | 7 | Describe all information sources in the search and date last searched. | Method – Literature Search |
| Search | 8 | Present full electronic search strategy for at least one database, including any limits used, such that it could be repeated. | Method – Literature Search |
| Study selection | 9 | State the process for selecting studies. | Method – Literature Search, Inclusion and Exclusion Criteria |
| Data collection process | 10 | Describe method of data extraction from reports and any processes for obtaining and confirming data from investigators. | Method – Data Extraction |
| Data items | 11 | List and define all variables for which data were sought and any assumptions and simplifications made. | Method – Data Extraction |
| Risk of bias in individual studies | 12 | Describe methods used for assessing risk of bias of individual studies, and how this information is to be used in any data synthesis. | Method – Data Extraction and Quality Assessment |
| Summary measures | 13 | State the principal summary measures. | Method –Statistical analysis |
| Synthesis of results | 14 | Describe the methods of handling data and combining results of studies, if done, including measures of consistency for each meta-analysis. | Method – Statistical analysis |

Page 1 of 2

| **Section/topic** | **#** | **Checklist item** | **Reported Section #** |
| --- | --- | --- | --- |
| Risk of bias across studies | 15 | Specify any assessment of risk of bias that may affect the cumulative evidence. | Method – Statistical analysis |
| Additional analyses | 16 | Describe methods of additional analyses, if done, indicating which were pre-specified. | Method – Statistical analysis |
| **RESULTS** | | |  |
| Study selection | 17 | Give numbers of studies screened, assessed for eligibility, and included in the review, with reasons for exclusions at each stage, ideally with a flow diagram. | Figure 1 |
| Study characteristics | 18 | For each study, present characteristics for which data were extracted and provide the citations. | Results – Demographics |
| Risk of bias within studies | 19 | Present data on risk of bias of each study and, if available, any outcome level assessment (see item 12). | Supplementary Table 3 |
| Results of individual studies | 20 | For all outcomes considered, present, for each study: (a) simple summary data for each intervention group (b) effect estimates and confidence intervals, ideally with a forest plot. | Results – All, Table1-2  Figure 2, 3 |
| Synthesis of results | 21 | Present results of each meta-analysis done, including confidence intervals and measures of consistency. | Results – Sexual Behaviors, Prevalence of HIV and Prevalence of Syphilis, HIV testing Behaviors, Figure 2, 3 |
| Risk of bias across studies | 22 | Present results of any assessment of risk of bias across studies (see Item 15). | Results –Sensitivity Analysis and Publication Bias |
| Additional analysis | 23 | Give results of additional analyses, if done (e.g., sensitivity or subgroup analyses, meta-regression [see Item 16]). | Results – Analysis of poppers use, Table 2 |
| **DISCUSSION** | | |  |
| Summary of evidence | 24 | Summarize the main findings including the strength of evidence for each main outcome; consider their relevance to key groups. | Discussion – Paragraph 1-7 |
| Limitations | 25 | Discuss limitations at study and outcome level, and at review-level. | Discussion – Limitations |
| Conclusions | 26 | Provide a general interpretation of the results in the context of other evidence, and implications for future research. | Discussion – Conclusions |
| **FUNDING** | | |  |
| Funding | 27 | Describe sources of funding for the systematic review and other support; role of funders for the systematic review. | This work was supported by the Mega-Projects of national science research (13rd Five-Year Plan [2017ZX10201101-002-007]), National Natural Science Foundation of China (81872674), National Science and Technology Major Project (2018ZX10101001-001-003) and Central Public-interest Scientific Institution Basal Research Fund of Chinese Academy of Medical Sciences (2018PT31042). |

*From:*  Moher D, Liberati A, Tetzlaff J, Altman DG, The PRISMA Group (2009). Preferred Reporting Items for Systematic Reviews and Meta-Analyses: The PRISMA Statement. PLoS Med 6(6): e1000097. doi:10.1371/journal.pmed1000097

Page 2 of 2
