# Supplementary material for: Association of nitrite inhalants use and unprotected anal intercourse and HIV/syphilis infection among MSM in China: a systematic review and meta-analysis
Source: BMC Public Health. 2020 Sep 10;20:1378. doi: 10.1186/s12889-020-09405-x (PMC7488293; doi:10.1186/s12889-020-09405-x)
Supplement: Supplementary file 2 — Additional file 2: Table S2. Checklist of quality assessment. It contains 5 questions to evaluate prevalence studies, decision criteria and scores for each question. [file 12889_2020_9405_MOESM2_ESM.doc]

**Supplementary Table 2. Checklist of quality assessment**

|  | **Question for Evaluating Prevalence Studies** | **Decision Criteria** | **Score** |
| --- | --- | --- | --- |
| Q1 | Was the sampling method representative of the population intended to the study? | Non-probability sampling (including: purposive, quota , convenience and snowball sampling) | 0 |
|  |  | Probability sampling (including: simple random, systematic, stratified, cluster, two-stage and multi-stage sampling) | 1 |
| Q2 | Was the measurement of HIV objective (if the article is focusing only on risk behaviour among MSM, please select “Not applicable” for this question)? | By questionnaires (Self-reported) | 0 |
|  |  | By clinical records or lab tests | 1 |
|  |  | Not applicable | NA |
| Q3 | Did the study report any response rate? (If the reported response rate is below 60%, the question should be answered “No”.) | No | 0 |
|  |  | Yes | 1 |
| Q4 | Did the investigator(s) control for confounding factors (e.g. stratification/ matching/ restriction/ adjustment) when analyzing the associations (if the study contains purely descriptive results, no association and prediction tests were conducted in the test, please select “Not applicable”)? | No | 0 |
|  |  | Yes | 1 |
|  |  | Not applicable | NA |
| Q5 | Was privacy or sensitivity of the nature of HIV considered when the survey was conducted eg if conducted in a non-MSM or general clinic setting? | No | 0 |
|  |  | Yes | 1 |
