# Supplementary material for: Association of nitrite inhalants use and unprotected anal intercourse and HIV/syphilis infection among MSM in China: a systematic review and meta-analysis
Source: BMC Public Health. 2020 Sep 10;20:1378. doi: 10.1186/s12889-020-09405-x (PMC7488293; doi:10.1186/s12889-020-09405-x)
Supplement: Supplementary file 3 — Additional file 3: Table S3. Summary of quality assessment score of 15 studies. The 15 studies were assessed on the quality assessment which include each score of five questions and total score of each study. [file 12889_2020_9405_MOESM3_ESM.doc]

**Supplementary Table 3. Summary of quality assessment score of 15 studies**

| **Study** | **Q1** | **Q2** | **Q3** | **Q4** | **Q5** | **QATSO Score** |
| --- | --- | --- | --- | --- | --- | --- |
| Chen et al. (2016) | 0 | NA | 1 | 1 | 1 | 75% |
| Chu et al. (2013) | 0 | 1 | 0 | 1 | 1 | 60% |
| Dong (2017) | 0 | 1 | 1 | 1 | 1 | 80% |
| Duan et al. (2017) | 0 | 1 | 1 | 1 | 1 | 80% |
| Huang et al. (2016) | 0 | 1 | 1 | 0 | 1 | 60% |
| Li (2014) | 0 | 1 | 1 | 1 | 1 | 80% |
| Li et al. (2014) | 0 | 1 | 1 | 1 | 1 | 80% |
| Li et al. (2016) | 0 | 1 | 1 | 1 | 1 | 80% |
| Wang (2016) | 0 | 1 | 1 | 0 | 1 | 60% |
| Wang et al. (2015) | 0 | NA | 1 | 0 | 1 | 50% |
| Wang et al. (2017) | 0 | 1 | 0 | 1 | 1 | 60% |
| Xu et al. (2017) | 0 | 1 | 0 | 1 | 1 | 60% |
| Yang et al. (2016) | 0 | 0 | 1 | 1 | 1 | 60% |
| Zhang et al. (2016) | 0 | 1 | 1 | 1 | 1 | 80% |
| Zhao et al. (2017) | 1 | 0 | 1 | 1 | 1 | 80% |
