# Supplementary material for: Association of nitrite inhalants use and unprotected anal intercourse and HIV/syphilis infection among MSM in China: a systematic review and meta-analysis
Source: BMC Public Health. 2020 Sep 10;20:1378. doi: 10.1186/s12889-020-09405-x (PMC7488293; doi:10.1186/s12889-020-09405-x)
Supplement: Supplementary file 4 — Additional file 4: Table S4. Basic information about eligible studies. Basic information on 15 eligible studies such as study location, study periods, recruitment methods, survey method, eligibility of subjects, sample size, recall window (months), poppers use recall window (months). [file 12889_2020_9405_MOESM4_ESM.doc]

**Supplementary Table 4.** Basic information about eligible studies

| **Study** | **Study location** | **Study periods** | **Recruitment methods** | **Survey method** | **Eligibility of subjects** | **Sample size** | **Recall window**  **(months)** | **Poppers use recall window**  **(months)** |
| --- | --- | --- | --- | --- | --- | --- | --- | --- |
| Chen et al.  (2016) | Guangdong Province | Mar to Aug, 2014 | gay website recruitment | self-administrated questionnaire | male over 16 years; had ever had anal sex with a male; studied in Guangdong Province | 825 | 3 | 3 |
| Chu  (2013) | Shenyang, Liaoning Province | Jan, 2011 to Dec, 2012 | respondent-driven sampling | face-to-face interview | 16 years or older; males who have had anal or oral sex with another male in the past 6 months | 625 | 3 | 3 |
| Dong  (2017) | Shanghai & Tianjin | Jan to Dec, 2016 | community outreach, the Internet intervention, and peer education | face-to-face interview | male; ≥18 years old; having had sexual contact (oral or anal sex) with other males in the past 6 months | 1365 | 6 | 6 |
| Duan et al.  (2017)^a^ | Shenzhen, Guangdong Province | Mar to Dec, 2015 | the time-location-sampling, the respondent driven sampling, and the snowball sampling | self-administrated questionnaire | being biologically male; having had sexual contact (oral or anal sex) with other males in Shenzhen during the past 6 months | 1935 | 6 | 6 |
| Huang et al.  (2016) | Xi'an, Shanxi Province | Apr to Jul, 2014, Sept to Dec, 2014, and Apr to Jul, 2015 | snowball sampling and venue-based mobilization | face-to-face interview | ≥16 years old; had anal or oral sex with other men in the past year | 1841 | 6 | 6 |
| Li  (2014) | 7 cities | Sept to Dec, 2012 | convenient sampling | face-to-face interview | ≥18 years; self-reported having anal intercourse with other men in the past 6 months; local residence for more than six months | 2806 | 6 | 6 |
| Li et al.  (2014)^a^ | Beijing | Jul to Oct, 2012 | website advertisement, community outreach, peer referrals, VCT | self-administrated questionnaire | at least 18 years old; self-reported ever having sex with men during the past 3 months | 400 | 3 | 3 |
| Li et al.  (2016) | Beijing | Apr to Dec, 2013 | short message service, web advertisement, community outreach, peer referral | face-to-face interview | males at least 18 years old; had sex with men in the last 12 months; currently living in Beijing | 1206 | 3 | 3 |
| Wang  (2016) | Kunming, Yunnan Province | 2015 | snowball sampling and online recruitment | self-administrated questionnaire | aged >18 years; ever had anal or oral sex with men | 635 | 6 | 6 |
| Wang et al.  (2015)^a^ | Beijing | Dec, 2012 to Jul, 2013 | outreaching in gay venues, website advertisement, peer referral | face-to-face interview | men age 18–60 years old living in Beijing; anal intercourse with at least one man in the last three months | 576 | 3 | 3 |
| Wang et al.  (2017)^a^ | Beijing & Nanjing, Jiangsu Province | Apr,2013 to Apr,2014 | online advertising and peer referral | face-to-face interview | 18 years or older; had anal intercourse at least once with another male in the past 12 months; currently living in Beijing or Nanjing | 510 | 6 | 6 |
| Xu et al.  (2017) | Nanjing, Jiangsu Province | Nov,2014 to Feb,2015 and Apr to Jul, 2015 | community organization mobilization, venue-based sampling, Internet recruitment ads, VCT | face-to-face interview | ≥16 years old; had anal or oral sex with other men in the past year; living in Nanjing | 1040 | 6 | 6 |
| Yang et al.  (2016)^a^ | Hunan Province | Jul, 2012 to Jan, 2013 | respondent-driven sampling via gay-dating website | self-administrated questionnaire | males aged >16 years; had provided sex services (receptive and/or penetrative) to other males for a fee in the previous 3 months | 205 | 3 | 3 |
| Zhang et al.  (2016)^a^ | Beijing | Apr, 2013 to Apr, 2014 | convenience sampling with four measures: short message service, community outreach, web advertisement, peer referral | face-to-face interview | males at least 18 years old; self-reported sex with men in the last 12 months; currently living in Beijing | 3588 | 3 | 3 |
| Zhao et al.  (2017)^a^ | nation-wide | Sept to Oct, 2014 | online recruitment | self-administrated questionnaire | being born as male; at least 16 years old; had ever engaged in anal sex with another man | 1424 | 12 | 12 |

^a^ The study was published in English.
